# Comparative proteomic analysis highlights metabolic dysfunction in α-synucleinopathy

**DOI:** 10.1038/s41531-020-00143-w

**Published:** 2020-12-11

**Authors:** Souvarish Sarkar, Michael A. Murphy, Eric B. Dammer, Abby L. Olsen, Srikant Rangaraju, Ernest Fraenkel, Mel B. Feany

**Affiliations:** 1 Department of Pathology, Brigham and Women’s Hospital, Harvard Medical School, Boston, MA USA; 2 Department of Biological Engineering, Massachusetts Institute of Technology, Cambridge, MA USA; 3 Department of Neurology, Emory University, Atlanta, GA USA; 4 Department of Neurology, Brigham and Women’s Hospital, Harvard Medical School, Boston, MA USA

**Keywords:** Parkinson's disease, Parkinson's disease

## Abstract

The synaptic protein α-synuclein is linked through genetics and neuropathology to the pathogenesis of Parkinson’s disease and related disorders. However, the mechanisms by which α-synuclein influences disease onset and progression are incompletely understood. To identify pathogenic pathways and therapeutic targets we performed proteomic analysis in a highly penetrant new *Drosophila* model of α-synucleinopathy. We identified 476 significantly upregulated and 563 significantly downregulated proteins in heads from α-synucleinopathy model flies compared to controls. We then used multiple complementary analyses to identify and prioritize genes and pathways within the large set of differentially expressed proteins for functional studies. We performed Gene Ontology enrichment analysis, integrated our proteomic changes with human Parkinson’s disease genetic studies, and compared the α-synucleinopathy proteome with that of tauopathy model flies, which are relevant to Alzheimer’s disease and related disorders. These approaches identified GTP cyclohydrolase (GCH1) and folate metabolism as candidate mediators of α-synuclein neurotoxicity. In functional validation studies, we found that the knockdown of *Drosophila* Gch1 enhanced locomotor deficits in α-synuclein transgenic flies, while folate supplementation protected from α-synuclein toxicity. Our integrative analysis suggested that mitochondrial dysfunction was a common downstream mediator of neurodegeneration. Accordingly, Gch1 knockdown enhanced metabolic dysfunction in α-synuclein transgenic fly brains while folate supplementation partially normalized brain bioenergetics. Here we outline and implement an integrative approach to identify and validate potential therapeutic pathways using comparative proteomics and genetics and capitalizing on the facile genetic and pharmacological tools available in *Drosophila*.

## Introduction

Parkinson’s disease is the most common neurodegenerative movement disorder and is characterized neuropathologically by the accumulation of protein aggregates known as Lewy bodies and Lewy neurites within neuronal cell bodies and processes. The synaptic vesicle-associated protein α-synuclein is a major constituent of these intracellular inclusion bodies^1^. Deposition of α-synuclein into inclusion bodies in neurons and glia is the defining feature of not only Parkinson’s disease, but also a group of related disorders including dementia with Lewy bodies and multiple system atrophy. These diseases are commonly termed α-synucleinopathies. In a striking convergence of neuropathology and genetics, infrequent familial cases of Parkinson’s disease are caused by highly penetrant mutations in *SNCA*, the gene encoding α-synuclein. The first familial mutations described in α-synuclein were the A53T^2^ and A30P^3^ missense mutations. The E46K point mutant was subsequently described^4^. We now know that in addition to missense mutations, duplications, and triplications of the α-synuclein encoding gene locus can cause penetrant familial Parkinson’s disease^5–8^. Thus, increasing levels of wild-type α-synuclein can cause disease, supporting strategies to model Parkinson’s disease by expressing the wild-type form of the protein, as in the current studies.

Disease-causing mutations that affect the levels or sequence of α-synuclein correlate well with the presence of α-synuclein aggregation in postmortem brains from Parkinson’s disease patients. However, α-synuclein mutations are rare. Most genetic variation promoting the development and progression of Parkinson’s disease derives from loci with lower penetrance. Concerted efforts to identify such variants through genome-wide association studies (GWAS) have resulted in almost 100 loci implicated in the genetic risk for Parkinson’s disease^9^. Examination of the molecular function of the proteins encoded by these loci does not immediately suggest precisely how variants at these loci promote α-synuclein aggregation and toxicity.

Interestingly, an apparent gap also exists between the function of proteins implicated in Parkinson’s disease pathogenesis by GWAS and the wealth of prior experimentation defining the pathophysiological basis of the disorder. Motivated by early work noting the clinical similarities between recreational drug users exposed to the mitochondrial complex I toxin 1-methyl-4-phenyl-1,2,3,6-tetrahydropyridine^10^ and patients with Parkinson’s disease, a large body of experimental work has implicated mitochondrial dysfunction in toxicity to dopaminergic neurons in the disorder. More recently, rare familial forms of Parkinson’s disease linked to mutations in genes encoding the proteins parkin and PINK1, which control mitochondrial dynamics and function have been identified^11,12^.

There is therefore a critical need to understand the mechanisms by which increased levels of wild-type α-synuclein, or mutations affecting the coding region of the protein, exert toxicity in Parkinson’s disease, with the goal of developing a comprehensive understanding of disease pathogenesis to support rational drug design. Proteomic analysis represents a powerful approach for biological discovery. The development of high-resolution mass spectrometers coupled with quantitative labeling and multiplexing strategies has increased the utility of the approach^13^. Here we have further incorporated integration with human genetic data and comparative proteomics with a second neurodegenerative disease model to enhance our ability to define mechanisms mediating α-synuclein neurotoxicity. Our results highlight Gch1 and folate as critical factors controlling α-synuclein neurotoxicity through downstream effects on mitochondrial function.

## Results

### Quantitative proteomics of human α-synuclein transgenic *Drosophila*

**Fig. 1:**
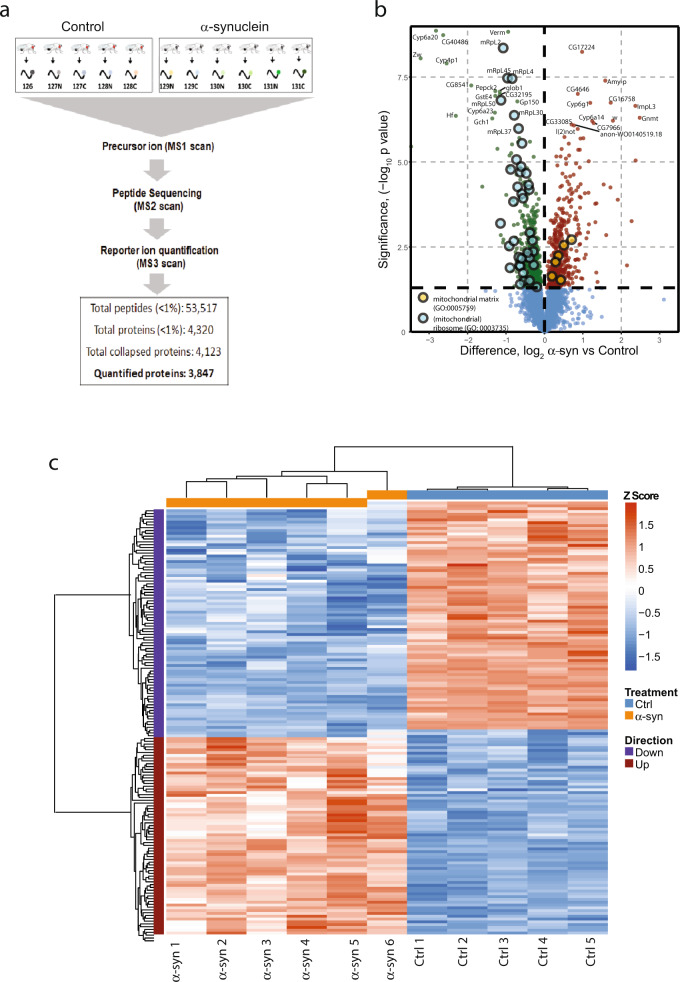
Quantitative proteomics of *Drosophila* expressing human α-synuclein. **a** Study outline for quantitative proteomics from fly heads using tandem mass tag mass spectrometry (TMT-MS). Protein extracts from five samples (ten heads each) of control and six samples (ten heads each) of wild-type human α-synuclein transgenic fly heads were digested, labeled with isobaric tags, and analyzed by MS. The total number of unique peptides and quantified proteins are shown. **b** Volcano plot showing results of differential expression analysis comparing control flies with human α-synuclein transgenic animals. The dashed vertical line differentiates the upregulated and downregulated proteins. The proteins above the dashed horizontal line represent the statistically significant proteins. The top GO terms for upregulated and downregulated proteins are highlighted. The identity of the top 30 differentially expressed proteins is provided. **c** Heatmap showing significantly altered proteins in α-synuclein transgenic flies. Supervised clustering of control and α-synuclein associated changes from 11-plex proteomic samples was performed with proteins differentially expressed below a Benjamini–Hochberg FDR of 0.01. Signal-to-noise ratio was transformed to *Z* scores, and clustering was performed on both proteins and samples using correlation distance with average metric. Control genotype: *nSyb-QF2, nSyb-GAL4/*+. Flies are 10 days old.

We recently described a *Drosophila* model relevant to Parkinson’s disease and related α-synucleinopathies in which human wild-type α-synuclein is expressed using the QF2 bipartite expression system^14^ and the *nSyb-QF2* pan-neuronal driver. Disease model flies show age-dependent locomotor dysfunction and neurodegeneration^15^, replicating key features of the human disorders. To investigate the mechanisms by which α-synuclein promotes neuronal toxicity we performed quantitative proteomics on heads from α-synuclein transgenic and control flies at 10 days of adult life (post eclosion) using tandem mass tag spectrometry (Fig. 1a). We identified 53,517 unique peptides, which mapped to 3847 proteins. Differential expression analysis revealed that 476 proteins were significantly upregulated, and 563 proteins were significantly downregulated in α-synuclein transgenic fly heads as illustrated with a volcano plot and hierarchical clustering (Fig. 1b, c, Supplementary Data Set 1). The majority of tissue in the fly head is of nervous system origin, but nonneuronal tissues such as muscle and fat body are also present and proteins from these organs may also be detected in our dataset.

We performed GO enrichment analysis on the differentially expressed proteins and found that proteins upregulated in α-synuclein transgenic fly heads were enriched for oxidation–reduction processes, oxidative stress, and electron transport chain function, while proteins downregulated in α-synuclein transgenic flies were enriched for mitochondrial ribosomal proteins and plasma membrane proteins (Fig. 1b, Supplementary Fig. 1a, b). Next, we performed network analysis using STRING^16^ on the α-synuclein upregulated and downregulated proteins separately to identify the KEGG pathways enriched with expression of human α-synuclein. Significantly enriched KEGG pathways included the pentose phosphate pathways and one-carbon pool of folate, and fatty acid biosynthesis (upregulated), TCA cycle, folate biosynthesis, and fatty acid metabolism (downregulated), among others (Supplementary Fig. 2a, b).

### Integration with human Parkinson’s disease genetics

**Fig. 2:**
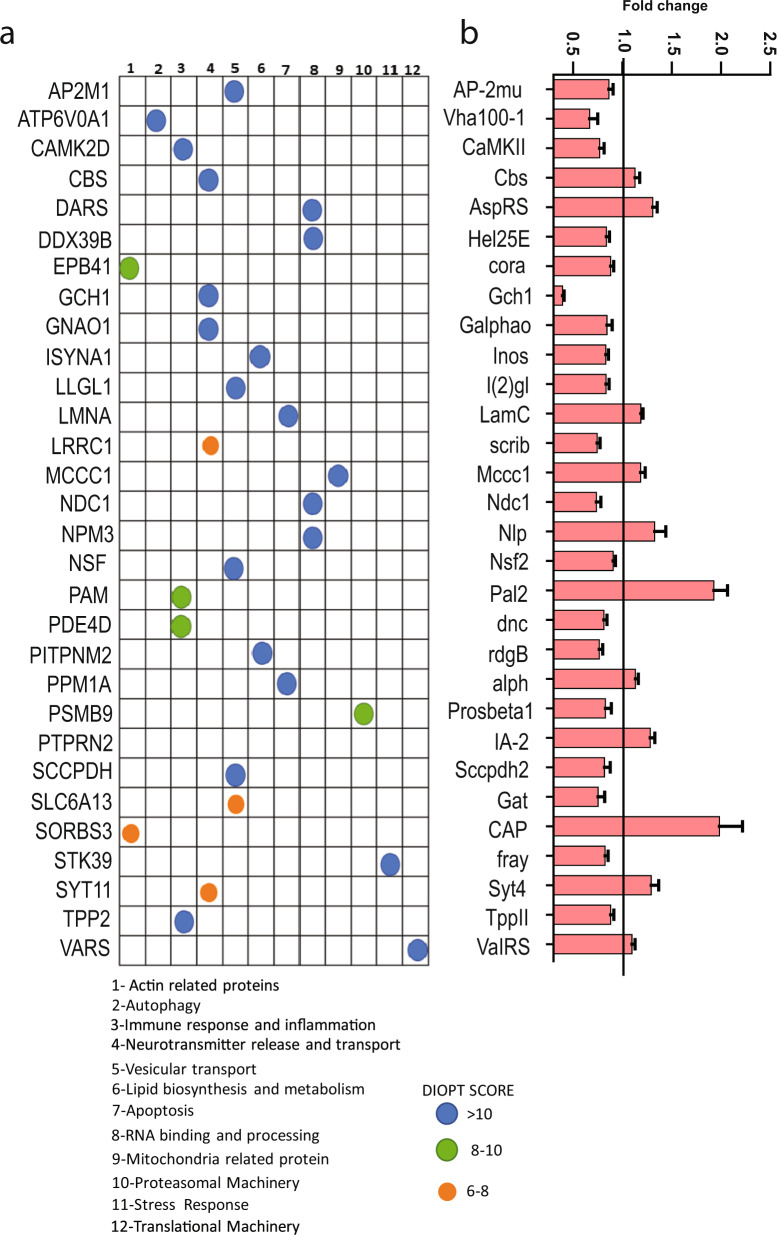
Human orthologs of differentially expressed proteins linked to Parkinson’s disease. **a** Human orthologs of significantly differentially expressed *Drosophila* proteins that have been implicated in Parkinson’s disease through GWAS. Proteins are organized into 12 categories based on functional data provided by STRING. The degree of homology is indicted by the DIOPT score (blue, green, or orange filled circles). **b** Normalized levels of the *Drosophila* proteins in α-synuclein transgenic flies compared to controls. The solid black line indicates the control level of the protein. All the proteins plotted are significantly differentially expressed, *p* < 0.05, two-tailed *t*-test. All data are represented as mean ± SEM.

Since the total number of proteins and pathways misregulated in human α-synuclein transgenic flies was large (Fig. 1, Supplementary Figs. 1, 2, Supplementary Data Sets 1,3) to identify key genes and pathways for mechanistic investigation we performed a comparative analysis using patient genetic and our *Drosophila* proteomic data. We cross-referenced our fly proteomic dataset with our previously published meta-analysis of existing Parkinson’s disease GWAS studies using gene-level association values calculated by MAGMA, which accounts for linkage disequilibrium using reference data with similar ancestry^17,18^. We used DIOPT^19^ to identify human orthologs of proteins altered in α-synuclein transgenic flies. We then compared these human orthologs to the MAGMA Parkinson’s disease genes, identifying 47 human orthologs of differentially expressed *Drosophila* proteins (Supplementary Data Set 2). Human orthologs with the top 30 DIOPT homology scores are shown in Fig. 2a. We performed a manual database curation using STRING network associations to assign these proteins to 12 functional categories, which include inflammation, neurotransmitter release, endosomal trafficking, actin dynamics, autophagy, and apoptosis (Fig. 2a). These processes have previously been linked to Parkinson’s disease pathogenesis^12,17,20–23^. Of the 30 proteins shown, 19 were downregulated while 11 were upregulated in α-synuclein transgenic flies (Fig. 2b).

### Comparative proteomics: *Drosophila* tauopathy

We next compared the fly α-synucleinopathy proteome with that of tauopathy model flies. In addition to *Drosophila* α-synucleinopathy models^15,24^, we have previously described models relevant to Alzheimer’s disease and related tauopathies in *Drosophila*. When we express wild-type or familial tauopathy mutant forms of human tau in flies we recapitulate key features of the disorders including behavioral deficits, neurodegeneration, and protein aggregation^25–27^. Expression of R406W mutant tau, linked to the neurodegenerative tauopathy frontotemporal dementia with parkinsonism linked to chromosome 17 (FTDP-17), produces levels of neurotoxicity amenable to genetic manipulation and we have thus focused significant effort towards characterization of the tau^R406W^ model^25,26,28–35^. As part of these studies we recently performed a proteomic analysis on heads of flies expressing tau^R406W^ using the pan-neuronal *elav-GAL4* driver at the same 10-day time point as analyzed in α-synuclein transgenic flies, using the same proteomics methodology (M.M., M.B.F., E.F., in preparation).

**Fig. 3:**
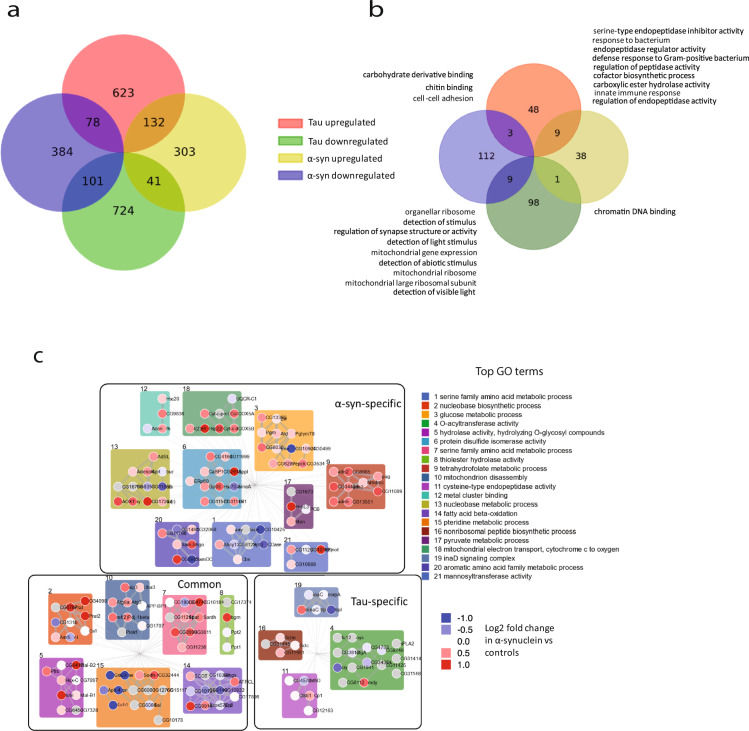
Human tau and α-synuclein expression induce distinct proteomic signatures. **a** Comparative proteomic analysis of human α-synuclein transgenic flies with flies expressing mutant human tau^R406W^ reveals largely distinct protein regulation. **b** Gene Ontology analysis of proteins that are significantly altered in both tau and α-synuclein transgenic flies. **c** Network analysis of proteins altered in tau and α-synuclein transgenic flies. Clusters are shaded by the color of the most-enriched GO term as defined on the right. The number on top of each cluster represents the GO term annotated in Table 1. Nodes corresponding to the indicated GO term are shaded to indicate degree of upregulation (red) or downregulation (blue). Edges are sized by confidence; edges across clusters are omitted for ease of visualization.

Comparison of the *Drosophila* α-synucleinopathy and tauopathy datasets revealed that proteins with altered expression in the two neurodegenerative disease models were largely distinct (Fig. 3a), consistent with the many clinical and neuropathological differences between α-synucleinopathies and tauopathies. Some proteins showed shared regulation: 132 of the 476 proteins upregulated in α-synuclein flies were also upregulated in human tau transgenic flies, while 101 of the 563 downregulated proteins in α-synuclein expressing flies were also reduced in human tau transgenic flies (Fig. 3a). GO analysis was performed to characterize shared and distinct pathways. Interestingly, mitochondrial function and immune response pathways were altered in both disease models (Fig. 3b). Both of these pathways have been implicated in α-synucleinopathy and tauopathy pathogenesis previously by our group and others^17,36,37^.

Because the number of pathways identified was large, particularly the α-synuclein and tau-specific pathways, we next performed network analysis to cluster both shared and distinct pathways. We used the solution of the prize-collecting Steiner forest algorithm (PCSF)^38^ to map proteomic alterations onto a network of physical protein interactions using *Drosophila* interactome data. Four separate PCSFs were constructed and Louvain community detection^39^ on the union of the α-synuclein and tau nodes was used to identify 21 final clusters. As expected from the GO analysis presented in Fig. 3b, clusters identified by network analysis revealed shared and distinct biological pathways (Fig. 3c). The most frequently identified pattern was specific for α-synuclein transgenic flies, with ten clusters. Clusters modified in both α-synuclein transgenic flies and tau transgenics consisted of seven clusters, while there were four tau-specific clusters. Each cluster was annotated for the most highly enriched GO term, revealing a range of metabolic and cytoskeletal processes (Fig. 3c, Table 1). These pathways represent a useful resource for further investigation of shared and distinct pathological processes in α-synucleinopathies and tauopathies.

**Table 1:** Top Gene Ontology annotations for each cluster in Fig. 3c.

Cluster	GO term	Specificity	FDR	Significant at FDR < 0.1?
1	Serine family amino acid metabolic process	α-syn	4.36E−03	TRUE
2	Nucleobase biosynthetic process	α-syn, tau	2.38E−05	TRUE
3	Glucose metabolic process	α-syn	6.85E−06	TRUE
4	O-acyltransferase activity	tau	6.62E−05	TRUE
5	Hydrolase activity, hydrolyzing O-glycosyl compounds	α-syn, tau	1.17E−03	TRUE
6	Protein disulfide isomerase activity	α-syn	3.85E−01	FALSE
7	Serine family amino acid metabolic process	α-syn, tau	5.40E−08	TRUE
8	Thiolester hydrolase activity	α-syn, tau	2.25E−02	TRUE
9	Tetrahydrofolate metabolic process	α-syn	2.16E−07	TRUE
10	Mitochondrion disassembly	α-syn, tau	8.15E−03	TRUE
11	Cysteine-type endopeptidase activity	tau	1.63E−03	TRUE
12	Metal cluster binding	α-syn	7.27E−04	TRUE
13	Nucleobase metabolic process	α-syn	8.50E−04	TRUE
14	Fatty acid beta-oxidation	α-syn, tau	1.46E−04	TRUE
15	Pteridine metabolic process	α-syn, tau	2.31E−04	TRUE
16	Nonribosomal peptide biosynthetic process	tau	5.36E−03	TRUE
17	Pyruvate metabolic process	α-syn	5.74E−01	FALSE
18	Mitochondrial electron transport, cytochrome c to oxygen	α-syn	4.52E−09	TRUE
19	inaD signaling complex	tau	9.36E−12	TRUE
20	Aromatic amino acid family metabolic process	α-syn	4.67E−04	TRUE
21	Mannosyltransferase activity	α-syn	1.93E−02	TRUE

GO analysis of the proteins in each cluster identified by comparative network analysis in α-synuclein and tau transgenic flies.

### Integrative analysis highlights Gch1 in α-synucleinopathy

**Fig. 4:**
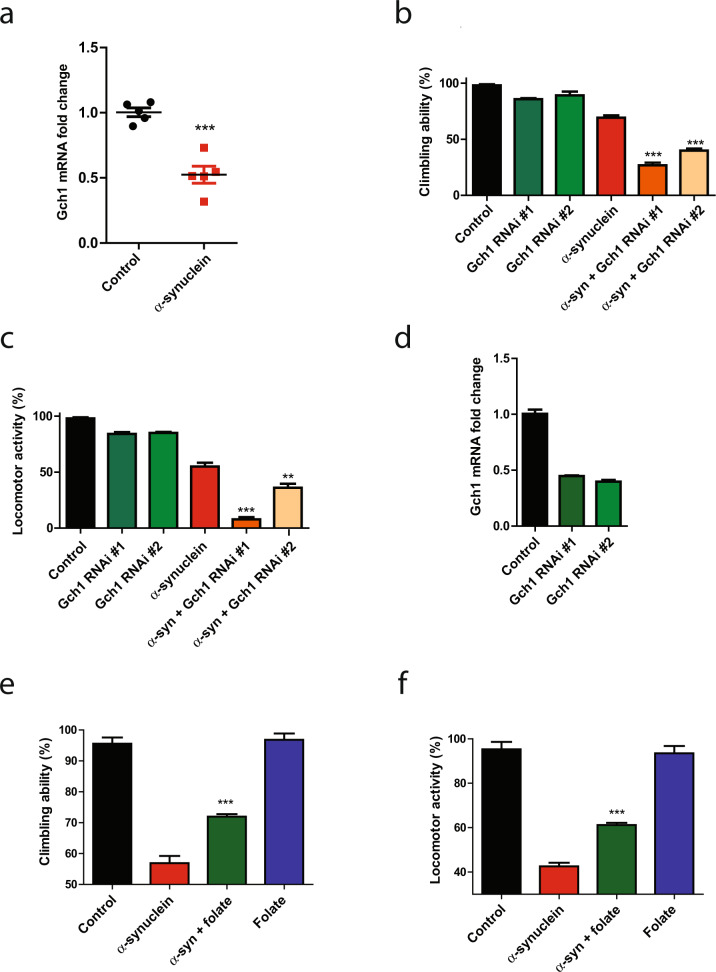
Gch1 and folate control α-synuclein toxicity. **a** Quantitative RT-PCR analysis shows decreased *Gch1* expression in human α-synuclein transgenic flies at 10 days of age when compared to control flies. Neuronal knockdown of Gch1 enhances α-synuclein induced (**b**) climbing and (**c**) locomotor activity deficits when compared to flies expressing α-synuclein alone. **d** Quantitative RT-PCR analysis shows the knockdown level of *Gch1* in the two RNAi lines used. Dietary folate supplementation in flies partially rescues α-synuclein induced (**e**) climbing and (**f**) locomotor activity deficits when compared to flies expressing α-synuclein not receiving folate. Control genotype: *nSyb-QF2, nSyb-GAL4/*+. All data are represented as mean ± SEM. For more than two groups, data were analyzed using one-way ANOVA with Tukey post-hoc test for multiple groups. For two groups, data are analyzed using two-tailed student’s *t* test. *p < 0.05, **p < 0.01, ***p < 0.005, *n* = 6. Flies are 10 days old in **a**–**c** and **e**, **f**, and 1 day old in **d**.

We hypothesized that our integrative analysis could identify functionally important genes and pathways in α-synucleinopathy pathogenesis. We thus examined the overlap of our proteomic (Fig. 1), human genetic (Fig. 2), and comparative network (Fig. 3) studies. One of the most highly downregulated proteins in our proteomic data set was GTP cyclohydrolase I (Gch1, also known in *Drosophila* as Punch), which is encoded by a gene orthologous to the human Parkinson’s disease GWAS locus *GCH1* (Fig. 2, Supplementary Data Set 2). Comparative network analysis identified the cluster containing Gch1 as a common cluster in tau and α-synuclein transgenic flies (Fig. 3c). Although the cluster was annotated as common by our informatic analysis, closer examination of the cluster revealed that multiple members of the cluster, including Gch1, were downregulated in α-synuclein expressing flies while those proteins were either not altered or upregulated in tau transgenic flies (Supplementary Fig. 3a), suggesting distinct modes of regulation in the two disease models. Consistent with distinct regulation, network analysis using up and downregulated proteins separately, identified a cluster containing Gch1 specific to α-synuclein transgenic animals (Supplementary Fig. 3b). Quantitative real-time PCR confirmed downregulation of *Gch1* in human α-synuclein transgenic flies (Fig. 4a). The mechanisms underlying the transcriptional alteration of *Gch1* in response to transgenic human α-synuclein expression remain to be determined, although regulation by nuclear α-synuclein is one possibility^1–4^.

Since proteomic, human genetic and network analysis all highlighted Gch1 as a strong candidate for further investigation in α-synucleinopathy we proceeded to functional testing. We first used transgenic RNAi to reduce Gch1 in our α-synuclein transgenic flies using the pan-neuronal *nSyb-GAL4* driver. Neuronal knockdown of Gch1 using either of two independent RNAi lines enhanced the motor deficits caused by α-synuclein expression, as shown by climbing (Fig. 4b) and locomotor activity (Fig. 4c). We verified that each RNAi line effectively reduced Gch1 expression (Fig. 4d). Importantly, knocking down Gch1 with transgenic RNAi had no effect on locomotion in the absence of human α-synuclein expression (Fig. 4b, c), using these partial loss of function lines (Fig. 4d), or on the levels of transgenic human α-synuclein (Supplementary Fig. 4).

### Integrative analysis suggests folate as a potential therapy in α-synucleinopathy

Although our analysis of Gch1 demonstrated the ability of our integrative approach to identify modifiers of α-synuclein neurotoxicity, significantly more work will be required to develop clinical treatments based on GCH1. Since an important goal of our work is to identify effective therapies for Parkinson’s disease, we examined our data for pathways potentially amenable to pharmacological manipulation. KEGG analysis identified folate biosynthesis as significantly downregulated (Supplementary Fig. 2a) and one-carbon pool by folate as significantly upregulated (Supplementary Fig. 2b) in α-synuclein transgenic flies. We therefore examined pathways regulating folate metabolism more closely in our proteomic dataset. Visualization with Cytoscape^40^ revealed that multiple proteins in the folate biosynthesis pathway were decreased in α-synuclein transgenic flies (Supplementary Fig. 5a, highlighted in yellow). In contrast, members of one-carbon pool by folate are upregulated (Supplementary Fig. 5b, highlighted in yellow). Since proteins in the folate biosynthetic pathway were decreased and proteins in folate utilization pathways were increased we hypothesized that folate supplementation might ameliorate α-synuclein neurotoxicity. We therefore fed α-synuclein transgenic flies *Drosophila* culture medium supplemented with 5 mM folate for 10 days. Flies treated with folate showed significantly improved locomotion as seen by improved climbing (Fig. 4e) and locomotor activity (Fig. 4f). Folate supplementation did not act by simply reducing the levels of transgenic α-synuclein as determined by western blot analysis (Supplementary Fig. 4).

### Gch1 and folate control bioenergetics in α-synucleinopathy model flies

**Fig. 5:**
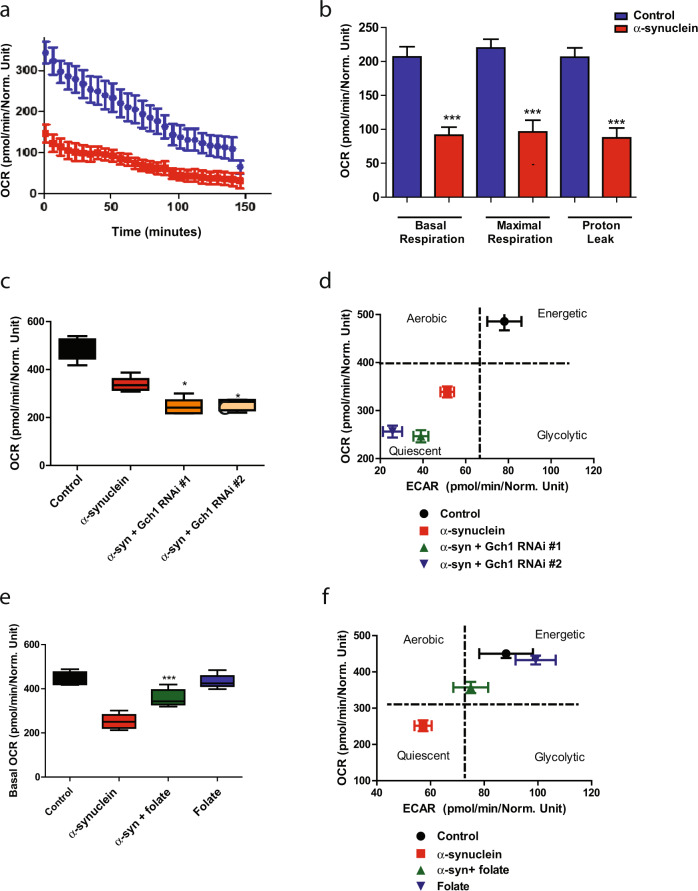
Gch1 and folate regulate bioenergetics in α-synucleinopathy model flies. Metabolic profiling of whole *Drosophila* brains in Seahorse XFe96-well culture microplates (Agilent) reveals decreased oxygen consumption rate (**a**, OCR), basal respiration, maximal respiration, and proton leak (**b**). **c** Neuronal knockdown of Gch1 further suppresses the basal oxygen consumption rate reduction in α-synuclein transgenic flies when compared to flies expressing α-synuclein alone. **d** The energy map reveals that neuronal knockdown of Gch1 enhances the whole brain energy deficit induced by α-synuclein expression. **e** Dietary folate supplementation partially normalizes the basal oxygen consumption rate reduction in α-synuclein transgenic flies when compared to flies expressing α-synuclein not receiving folate. **f** The energy map shows that folate supplementation partially rescues the whole brain energy deficit induced by α-synuclein expression. Control genotype: *nSyb-QF2, nSyb-GAL4/*+. All data are represented as mean ± SEM. Data are analyzed using one-way ANOVA with Tukey post-hoc test for multiple groups. *p < 0.05, **p < 0.01, ***p < 0.005, *n* = 3–6. Flies are 10 days old.

We have previously shown that α-synuclein expression in our transgenic *Drosophila* leads to altered mitochondrial morphology and increased oxidative stress^15^. To assess the influence of altered Gch1 expression on metabolic function we used a method recently described for the analysis of intact fly brains in the Seahorse XFe96 Analyzer^41^. We found that α-synuclein transgenic flies had reduced basal respiration rate, maximal respiration, and proton leak (Fig. 5a, b). We then assessed metabolism in α-synuclein transgenic brains following RNAi-mediated reduction of Gch1 levels. We found that neuronal knockdown of Gch1 further reduced basal OCR in α-synuclein transgenic brains (Fig. 5c). Gch1 knockdown additionally promoted a more quiescent metabolic state in the brain (Fig. 5d). Knockdown of Gch1 in the absence of transgenic human α-synuclein expression, with the partial loss of function RNAi lines used here (Fig. 4d), had no effect on metabolism as measured in the Seahorse Analyzer (Supplementary Fig. 6a).

In contrast to Gch1 knockdown, folate supplementation partially restored the basal oxygen consumption rate (OCR) in α-synuclein transgenic flies (Fig. 5e). The energy map additionally revealed that folate supplementation promoted a more aerobic metabolic state (Fig. 5f). Again, folate supplementation did not alter metabolism in the absence of transgenic α-synuclein expression (Fig. 5e, f).

### Role of glial protein changes in α-synucleinopathy

**Fig. 6:**
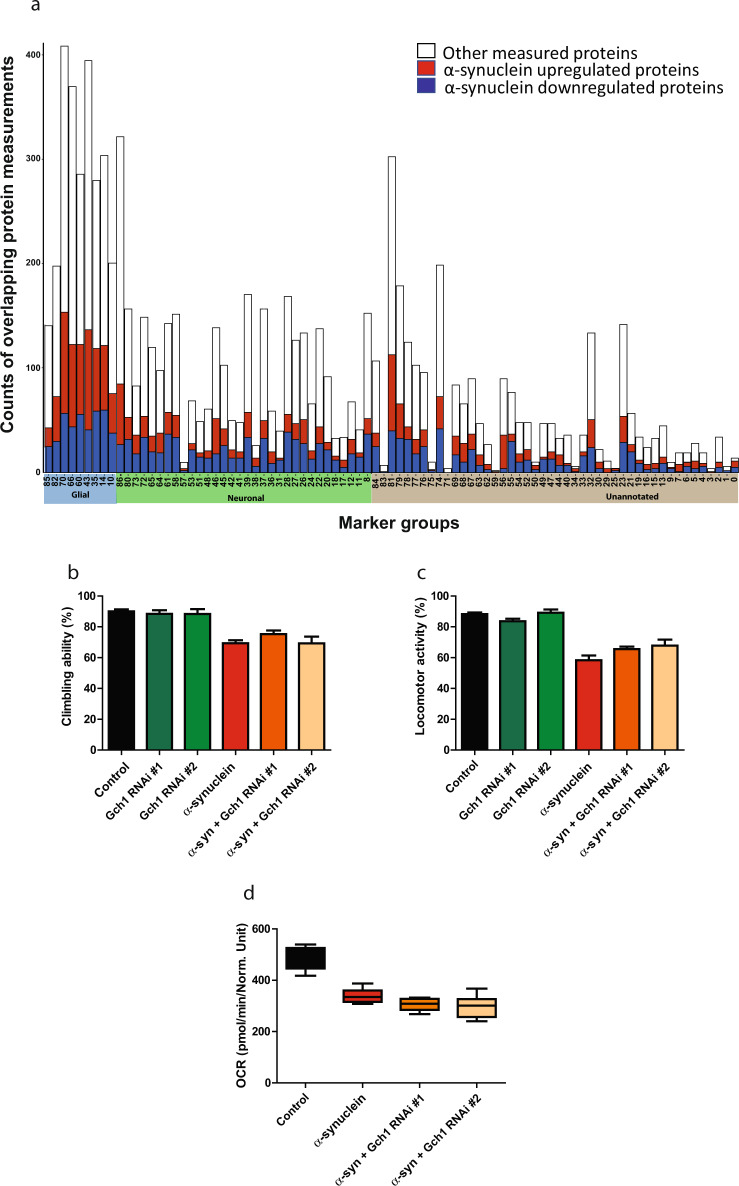
Cell type specific protein regulation in α-synucleinopathy. **a** Cell type distribution of proteins that are differentially expressed when human α-synuclein is expressed in fly neurons. Each bar shows the number of upregulated (red), downregulated (dark blue), and total measured (white) proteins within cell type subgroups. The clusters are separated into three groups: glial (highlighted in light blue on the *x*-axis), neuronal (highlighted in green on the *x*-axis), and unannotated (highlighted in gray on the *x*-axis)^42^. Glial knockdown of Gch1 does not modify α-synuclein induced (**b**) climbing deficits, (**c**) locomotor activity deficits, or (**d**) basal oxygen consumption rate reduction when compared to flies expressing α-synuclein alone. Control genotype: *nSyb-QF2, nSyb-GAL4/*+. All data are represented as mean ± SEM. Data are analyzed using one-way ANOVA with Tukey post-hoc test for multiple groups. *n* = 3–6. Flies are 10 days old.

We found that knockdown of Gch1 specifically in neurons enhanced α-synuclein toxicity. However, Gch1 is also expressed in glia. Analysis of a reference single-cell RNA sequencing study of the fly brain^42^ revealed that in addition to the expected expression in monoaminergic neurons, Gch1 is also present in glial cells (Supplementary Fig. 6b). A similar analysis of mouse and human data (Brain RNA-Seq, RRID:SCR_013736^43^) revealed glial expression of GCH1 as well (Supplementary Fig. 6c). To determine if other proteins with altered expression in our *Drosophila* α-synucleinopathy model were also present in nonneuronal cells, we mapped differentially expressed proteins to distinct neuronal, glial and unannotated clusters as identified by single-cell RNA sequencing^42^. Of the entire set of 3847 proteins we identified in the fly brain with our proteomic analysis (Fig. 1a), 3077 mapped to genes also identified in the reference single-cell RNAseq study. Genes identified in the RNAseq study with predicted proteins identified in our proteomic study included 1047 that were predominantly neuronal, 905 that were predominantly glial and 1125 unannotated, as classified by the RNAseq analysis^42^. Many genes were expressed in more than one cell type as determined by RNAseq analysis. For genes expressed in more than one cell type we assigned the gene to the cell type with the highest expression for the purposes of our subsequent analysis. Of 905 designated glial proteins, 175 were upregulated while 137 were downregulated when human α-synuclein was expressed in neurons. Interestingly, proteins upregulated when transgenic α-synuclein was expressed in neurons were more highly enriched for designated glial proteins (Fig. 6a, highlighted in blue) as compared to designated neuronal proteins (Fig. 6a, highlighted in green; Supplementary Data Set 4, Chi-square 16.8 *p* = 0.002). Glial proteins that were modulated by α-synuclein expression in neurons include Eaat1 and wrapper, both proteins with documented predominant glial localization and important functional roles in glia^44,45^.

Our new *Drosophila* α-synucleinopathy model was designed using the bipartite QF2 expression system^14^, rather than the more widely used GAL4/UAS system^46^, to allow simultaneous and independent manipulation of gene expression in neurons and glia^47,48^. To probe a potential functional role of glial Gch1 we knocked down the gene specifically in glia (*repo-GAL4* pan-glial driver), while expressing α-synuclein in neurons (*nSyb-QF2* pan-neuronal driver). Glial knockdown of Gch1 did not alter motor dysfunction in α-synuclein transgenic flies (Fig. 6b, c). Similarly, glial knockdown of Gch1 had no effect on whole brain metabolism as measured with the Seahorse XFe96 Analyzer (Fig. 6d). These data suggest that although Gch1 is expressed in both neurons and glia, neuronal function is critical for modification of α-synuclein toxicity, at least under the current experimental conditions.

## Discussion

Here we present a proteomic analysis of a new *Drosophila* model relevant to Parkinson’s disease and related α-synucleinopathies, which we have recently described^15^. Compared to our prior models of α-synucleinopathy, our new model shows accelerated locomotor dysfunction and more widespread neurodegeneration. The enhanced behavioral and neuropathological abnormalities in our new model plausibly correlate with higher levels of α-synuclein expression. However, α-synuclein is still expressed within a physiological range with levels per mg total protein comparable to human brain homogenate^5^. Increased toxicity may also reflect the use of the *nSyb-QF2* driver in the new model compared with the *elav-GAL4* driver used previously^6^. Consistent with the substantial biological toxicity in our new model, we find altered regulation of a large number of proteins (Fig. 1b) in comparison to prior proteomic analyses in our earlier models^49,50^. An important rationale for performing proteomic analysis in a *Drosophila* model of Parkinson’s disease and related disorders is the ability to use the facile genetic and pharmacological tools available to test the causative role of changes identified by proteomics or other large-scale omics analyses^51^. However, the large number of differentially expressed proteins in our data set represents a challenge even for the higher throughput candidate testing possible in flies compared to vertebrate systems. In addition, our ultimate goal is translation back to the human disease we are modeling. Thus, we employed a multilayered strategy to prioritize candidates from our proteomic study for functional analysis.

We began by integrating our proteomic data with genetic risk factors for Parkinson’s disease defined by GWAS. We chose to use GWAS not only because the loci identified represent the best source of human genetic variation predisposing to the common neuropathology of α-synucleinopathy, but also because functional validation in genetic model organisms is highly complementary to the data generated by GWAS^20,52^. While GWAS provides invaluable causal evidence linking loci to Parkinson’s disease pathogenesis, additional work is often needed to pinpoint the exact gene mediating the effect and to understand the molecular mechanisms involved. Further, if an implicated gene is expressed in more than one potentially relevant cell type, GWAS data alone will not be sufficient to determine the cell type in which the variant exerts its effect.

In the case of GCH1, although a number of genetic studies have suggested that common variants in GCH1 are linked to Parkinson’s disease^9,17^, other studies have not found an association^53–55^. Our functional data provide complementary evidence for a role of GCH1 in Parkinson’s disease and other disorders characterized by abnormal aggregation and deposition of α-synuclein.

Our results further suggest a previously unsuspected role for Gch1 in controlling brain energy metabolism. GCH1 catalyzes rate-limiting step in the tetrahydrobiopterin (BH4) biosynthesis. BH4 is a critical cofactor for the synthesis of monoamine neurotransmitters, including dopamine. Loss of function mutations in GCH1 cause the rare disorder dopa-responsive dystonia, which is managed clinically with oral dopamine replacement therapy. Given the prominent role of dopamine neuronal loss in Parkinson’s disease, a shared pathogenesis related to abnormal dopamine metabolism has been speculated between dopa-responsive dystonia and Parkinson’s disease. Our data alternatively suggest that GCH1 may play a more general role in energy metabolism. In addition to a critical role in regulating monoamine production through tyrosine hydroxylase, BH4 is also an essential cofactor for nitric oxide synthase. In the cardiovascular system, BH4 has been implicated in the regulation of redox through regulation of endothelial and macrophage nitric oxide synthase^56^. Control of mitochondrial metabolism via nitric oxide synthase independent mechanisms, including effects through the key transcriptional regulator of mitochondrial biogenesis and function PGC-1α, have also been reported^57,58^. One or more of these mechanisms may account for the enhancement of brain bioenergetic dysfunction by Gch1 knockdown we observe in our *Drosophila* α-synucleinopathy model.

Alternatively, mitochondrial pathology could be downstream of disrupted aminergic signaling. As in mammalian systems, monoaminergic neurons in *Drosophila* have widespread projections throughout the brain^59^. Similarly, receptors for dopamine and other monoamines are present on many neurons and glia, allowing for second messenger mediated influence of mitochondrial biogenesis and function^60–62^. Additional work will be required to distinguish these possible modes of Gch1 action in controlling energy metabolism.

Although our data do not identify the specific mechanism by which Gch1 influences cellular bioenergetics, our findings do suggest that Gch1 acts in neurons, not glia to provide neuroprotection. Our new model of α-synucleinopathy was designed to allow simultaneous but independent control of gene expression in neurons and glia so that neuronal and glial influences on neurodegeneration could be dissected genetically. We have previously used our system to investigate the transcriptional and neuropathological effects of expressing α-synuclein in neurons, glia, or both cell types^47^. Here we show that although Gch1 is expressed in both neurons and glia in flies (Supplementary Fig. 6b), mice (Supplementary Fig. 6c) and humans (Supplementary Fig. 6c), knockdown of Gch1 in neurons, but not glia, worsens the bioenergetic deficit created by expression of α-synuclein in neurons (Figs. 5, 6). Thus, although the GWAS signal implicating GCH1 does not indicate the cell type responsible for the disease-modifying effect, our functional data are consistent with a critical role for GCH1 in neurons in influencing α-synucleinopathy pathogenesis.

Neurodegenerative disorders, including α-synucleinopathies and tauopathies, have a number of intriguing similarities. These diseases are all characterized by usual late adult onset, predilection for particular anatomic and cell types, and deposition of insoluble proteins aggregates within brain tissue. These common features, in particular the presence of protein aggregates, have led to the notion that underlying mechanisms might be similar in the disorders. In the case of invertebrate modeling there is also the possibility that expression of a foreign human protein might produce nonspecific toxicity, although we have ensured that expression levels of human α-synucleinopathy and tau are moderate, equivalent to normal brain levels^15,26^. Despite these theoretical and technical considerations, our prior genetic^63^ and gene expression^51^ analyses have suggested that pathological mechanisms are substantially distinct in fly models of neurodegenerative disease associated with toxic aggregating proteins. Our current data similarly show that the majority of proteins and pathways with altered expression in α-synucleinopathy and tauopathy model flies are distinct (Fig. 3a–c). Proteins and pathways that we identify as having distinct regulation in the two models, like Gch1, represent attractive candidates for additional mechanistic investigation in the future.

Our work identifies shared proteins and pathways as well (Fig. 3). Notably, mitochondrial metabolism is altered in both models. These findings fit well with prior data from our laboratory suggesting that mitochondrial dysfunction is a common downstream mechanism triggered by the interaction of α-synuclein and tau with distinct upstream binding partners^15,30,36^. More generally, work from many investigators has implicated mitochondrial dysfunction in the pathogenesis of Parkinson’s disease, Alzheimer’s disease, and related disorders^12,37,64^.

Brain bioenergetics is responsive to both Gch1 genetic manipulation and folate supplementation in α-synuclein transgenic flies (Fig. 5). We do not know if the molecular and cellular pathways mediating the effects of Gch1 and folate on mitochondrial metabolism are connected. Further work will be required to determine if and how the two are related. An interaction is plausible because folate can promote the expression of dihydrofolate reductase, which recycles BH2 back to BH4^65,66^. More generally, our results are consistent with prior studies implicating folate metabolism in the control of mitochondrial function and oxidative stress in *Drosophila*^67,68^, including in parkin^69^ and Pink1^70^ deficient flies. Despite significant interest in vitamins, including folate^71^, as treatments for Parkinson’s disease clinical efficacy of folate and other vitamins has not been shown. Our findings raise the possibility that folate supplementation might be particularly beneficial in patients with genetic or environmental deficiencies in BH4. More generally, the results presented here outline an approach to analyze and prioritize proteomic or other omic data using an integrative approach across species and experimental models, followed by in vivo functional validation.

## Methods

### *Drosophila* genetics and behavior

*Drosophila* crosses were performed at 25 °C. Flies were aged at 25 °C for 10 days. Equal numbers of male and female flies were used in each experiment. The pan-neuronal drivers *nSyb-GAL4* and *nSyb-QF2*, and the pan-glial *repo-GAL4* driver were used to mediate gene expression as detailed in the results and figure legends. Our laboratory has previously described *QUAS-wild-type α-synuclein*^15^ and *UAS-tau-R406W*^26^ transgenic flies. The following stocks were obtained from the Bloomington *Drosophila* Stock Center: *nSyb-GAL4*, *repo-GAL4*, *UAS-Gch1 (Pu) RNAi 1* (HMS02399, Bloomington #41998), and *UAS-Gch1 RNAi 2* (HMC04085, Bloomington #55397). Dr. Christopher Potter kindly provided the *nSyb-QF2* driver line. Flies were maintained on standard cornmeal-agar medium with the exception of folate supplementation experiments. Dietary supplementation with folate was performed as previously described^72^ with minor modifications. Folate was added to instant fly food (Carolina Biological) at a final concentration of 5 mM, based on previously effective oral folate concentrations in *Drosophila*^72^. Adult flies were added to food at 1 day after eclosion and were aged to 10 days. Drug-embedded food was changed every 3 days.

The climbing and locomotor activity assays were performed as previously described^25,47^. Briefly, for the climbing assay, the number of flies climbing 5 cm in 10 s were calculated. For the activity assay, the vials were tapped and placed horizontally for 15 s. After 15 s the number of flies moving was recorded. For both locomotor assays, ten flies were placed in each vial and six vials were used for each genotype. One-way ANOVA with Tukey post-hoc test was used for statistical analysis, with *n* = 6.

### Quantitative mass spectrometry

Five control (genotype: *nSyb-GAL4, nSyb-QF2 /*+*)* and six α-synuclein transgenic (genotype: *QUAS-wild-type α-synuclein, nSyb-GAL4, nSyb-QF2 /*+*)* samples of ten fly heads each were used for proteomic analysis. Samples were prepared as previously described^73^ with the following modifications. All solutions are reported as final concentrations. *Drosophila* heads were lysed by sonication and passaged through a 21-gauge needle in 8 M urea, 200 mM EPPS, pH 8.0, with protease and phosphatase inhibitors (Roche). Protein concentration was determined with a micro-BCA assay (Pierce). Proteins were reduced with 5 mM TCEP at room temperature for 15 min and alkylated with 15 mM Iodoacetamide at room temperature for 1 h in the dark. The alkylation reaction was quenched with dithiothreitol. Proteins were precipitated using the methanol/chloroform method. In brief, four volumes of methanol, one volume of chloroform, and three volumes of water were added to the lysate, which was then vortexed and centrifuged to separate the chloroform phase from the aqueous phase. The precipitated protein was washed with one volume of ice-cold methanol. The protein pellet was allowed to air dry. Precipitated protein was resuspended in 200 mM EPPS, pH 8. Proteins were digested with LysC (1:50; enzyme:protein) overnight at 25 °C followed by trypsin (1:100; enzyme:protein) for 6 h at 37 °C. Peptide quantification was performed using the micro-BCA assay (Pierce). Equal amounts of peptide from each sample was labeled with tandem mass tag (TMT10) reagents (1:4; peptide:TMT label) (Pierce). The 10-plex labeling reactions were performed for 2 h at 25 °C. Modification of tyrosine residues with TMT was reversed by the addition of 5% hydroxyl amine for 15 min at 25 °C. The reaction was quenched with 0.5% trifluoroacetic acid and samples were combined at a 1:1:1:1:1:1:1:1:1:1:1 ratio. Combined samples were desalted and offline fractionated into 24 fractions as previously described.

12 of the 24 peptide fractions from the basic reverse phase step (every other fraction) were analyzed with an LC-MS3 data collection strategy^74^ on an Orbitrap Lumos mass spectrometer (Thermo Fisher Scientific) equipped with a Proxeon Easy nLC 1000 for online sample handling and peptide separations. Approximately 5 µg of peptide resuspended in 5% formic acid + 5% acetonitrile was loaded onto a 100 µm inner diameter fused-silica micro capillary with a needle tip pulled to an internal diameter less than 5 µm. The column was packed in-house to a length of 35 cm with a C_18_ reverse phase resin (GP118 resin 1.8 μm, 120 Å, Sepax Technologies). The peptides were separated using a 180 min linear gradient from 3% to 25% buffer B (100% acetonitrile + 0.125% formic acid) equilibrated with buffer A (3% acetonitrile + 0.125% formic acid) at a flow rate of 600 nL/min across the column. The scan sequence began with an MS1 spectrum (Orbitrap analysis, resolution 120,000, 350–1350 *m/z* scan range, AGC target 1 × 10^6^, maximum injection time 100 ms, dynamic exclusion of 75 s). The “Top10” precursors were selected for MS2 analysis, which consisted of CID (quadrupole isolation set at 0.5 Da and ion trap analysis, AGC 1.5 × 10^4^, NCE 35, maximum injection time 150 ms). The top ten precursors from each MS2 scan were selected for MS3 analysis (synchronous precursor selection), in which precursors were fragmented by HCD prior to Orbitrap analysis (NCE 55, max AGC 1.5 × 10^5^, maximum injection time 150 ms, isolation window 2 Da, resolution 50,000).

A suite of in-house software tools was used for. RAW file processing and controlling peptide and protein level false discovery rates, assembling proteins from peptides, and protein quantification from peptides as previously described^74^. MS/MS spectra were searched against a Uniprot *Drosophila* reference database appended with common protein contaminants and reverse sequences. Database search criteria were as follows: tryptic with two missed cleavages, a precursor mass tolerance of 50 ppm, fragment ion mass tolerance of 1.0 Da, static alkylation of cysteine (57.02146 Da), static TMT labeling of lysine residues and N-termini of peptides (229.162932 Da), and variable oxidation of methionine (15.99491 Da). TMT reporter ion intensities were measured using a 0.003 Da window around the theoretical *m/z* for each reporter ion in the MS3 scan. Peptide spectral matches with poor quality MS3 spectra were excluded from quantitation (<200 summed signal-to-noise across 10 channels and <0.7 precursor isolation specificity).

Differences in protein expression data were determined using pairwise *t-*test applied to log2-transformed expression data for normalization. Gene Ontology (GO) enrichment analyses were performed using GO-Elite software as previously described^17^ using input lists of differentially expressed proteins that were either increased or decreased in α-synuclein expressing flies. GO-Elite uses a *Drosophila* database to perform GO enrichment. Pathway analyses were also performed (Metacore, Thompson Reuters) as previously described^75^. For GO enrichment analysis a *Z*-score of 2 was used a cutoff. For KEGG pathway enrichment, the differentially expressed proteins were used as input in STRING^16^ and the enriched KEGG pathways with FDR < 0.05 were considered significant.

### Comparative proteomics

Log-fold-changes and *p* values for each condition were computed as above. The Venn diagram indicates proteins with nominal *p* value < 0.05; statistical significance of overlaps was computed via Fisher’s exact test.

For gene set enrichment analysis log-fold-changes were passed separately for each condition into the “Prerank” function of a Python implementation of GSEA^76^. Gene sets used were GO terms: associations between fly UniProt IDs and GO terms were downloaded from UniProt^77^ on Mar 24, 2020 and were propagated using the “basic” ontology downloaded on Mar 27, 2020^78^, with terms labeled “obsolete” removed. The set of proteins detected in either experiment was used as background, and gene sets tested were restricted to those with size between 10 and 100. The Venn diagram indicates gene-sets with nominal *p* value < 0.05; statistical significance of overlaps was computed via Fisher’s exact test.

An interactome was constructed using STRING v11 for *Drosophila melanogaster*^16^, restricted to the largest connected component induced by including only physical-binding edges with “experimental”, “database”, “transferred experimental”, or “transferred database” evidence. Each protein was then identified as dysregulated in tau or synuclein if had an FDR-adjusted *p* value < 0.1. PCSF^38^ was then run separately upon each of those two sets of proteins as terminal nodes, weighted by the absolute value of log2-fold-change, clipped to ±1. The edge costs were set to 1.5 minus the STRING confidence score of each edge. The parameters used were prize-scaling factor β = 2 and hub penalty γ = 4. For each set of prize nodes PCSF was run 300 times with Gaussian jitter of σ = 0.1 added to edges; nodes detected in >100 of randomizations were kept. The specificity of these networks was confirmed via degree-preserving randomization of the terminals: no node appeared in more than 10% of random networks. The nodes arising from each prize set were then pooled; their induced subgraph was then extracted from the interactome and trimmed to the largest connected component.

To cluster the resulting graph into pathways, first PageRank^79^ with α = 0.85 was run two times, once for each prize set, with the corresponding weights as the personalization vector. This yields a length-2 vector of “smoothed” absolute log-fold-changes for each node, for each of tau and α-synuclein. For each pair of nodes joined by an edge, an “affinity” score was computed as the cosine of the angle between these vectors. This score approaches 1 when both nodes display the same pattern of differential expression between tau and synuclein, and 0 when they display the opposite. Louvain community detection^39^ was then run using these affinity scores as edge weights, using γ = 4. The solution with the best modularity score out of 100 random initializations was selected. One singleton cluster was discarded, yielding 22 clusters ranging between size 4 and 15. The final network comprised 173 nodes, 26 of which were Steiner nodes, and 271 edges. The visualization was generated using the “Axial” Python library. (http://alexlenail.me/Axial/).

Each cluster was then annotated with its most-enriched GO term (one-sided Fisher’s exact test) using the interactome as background and restricting to terms with between 5 and 50 proteins; all but two clusters reached FDR-adjusted significance <0.1. To assign specificity, clusters were labeled as dysregulated for each of tau and synuclein if more than 30% of their nodes had FDR < 0.1 in that condition.

### Measurement of oxygen consumption and extracellular acidification rates

The OCR and extracellular acidification rate were measured using a Seahorse XFe96 metabolic analyzer following the procedure recommended by the manufacturer. For all experiments, brains from 10-day-old flies of the appropriate genotypes were dissected and plated at one brain per well on XFe96 plates (Seahorse Bioscience) and metabolic parameters assayed as described^41^. The OCR values were normalized to DNA content using a CyQUANT assay (ThermoFisher) following the manufacturer’s protocol.

### Quantitative real-time PCR

For reverse transcription, RNA was extracted from four fly heads using QIAzol (QIAGEN). 1 µg of RNA was reverse transcribed using the Applied Biosystems High-Capacity cDNA Reverse Transcription Kit using the manufacturer’s protocol. SYBR Green (Applied Biosystems) based qPCR was performed on an Applied Biosystems QuantStudio 6 Flex Real-Time PCR System. Primer sequences for *Gch1* were GCACGCTCGTATCGTCTACT (forward) and AGACTCTGGTCGTAGCCCTT (reverse). Primer sequences for *RpL32*, used as a control, were GACCATCCGCCCAGCATAC (forward) and CGGCGACGCACTCTGTT (reverse). The fold change in gene expression was determined by the ΔΔC_t_ method, where C_t_ is the threshold value.

### Gel electrophoresis and immunoblotting

For denaturing polyacrylamide gel electrophoresis, *Drosophila* heads were homogenized in 2× sample buffer and the resulting homogenates analyzed on 4–20% precast gels (Bio-Rad) and immunoblotted according to standard protocols. All blots were repeated at least three times with similar results. Images of representative blots are shown in the figures. All blots presented in individual figures derive from the same experiment and were processed in parallel. Primary antibodies were used at the indicated concentrations: α-synuclein (H3C), 1:350,000, Developmental Studies Hybridoma Bank; GAPDH, 1:10,000, Abcam.

### Statistical analysis

For Figs. 4, 5, 6, and Supplementary Fig. 6 comparisons across more than two groups, one-way ANOVA with Tukey post analysis was used. For two groups students *t* test were performed using GraphPad prism 5.0. The Grubbs’ test was applied to data and outliers were not detected. Data collection was randomized.

### Reporting summary

Further information on research design is available in the Nature Research Reporting Summary linked to this article.

## Supplementary Information

Supplementary figures

Supplementary data 1

Supplementary data 2

Supplementary data 3

Supplementary data 4

Reporting summary

## Data Availability

Raw data supporting the results reported in this article are in the figure source data files, which are available upon request. The entire raw dataset for the α-synuclein proteomics is available in ProteomeXchange via the PRIDE database (Project accession: PXD021312).
