# Supplementary material for: Comparative proteomic analysis highlights metabolic dysfunction in α-synucleinopathy
Source: NPJ Parkinsons Dis. 2020 Dec 11;6:40. doi: 10.1038/s41531-020-00143-w (PMC7732845; doi:10.1038/s41531-020-00143-w)
Supplement: Supplementary file 1 — Supplementary figures [file 41531_2020_143_MOESM1_ESM.pdf]

Supplementary Figure 1

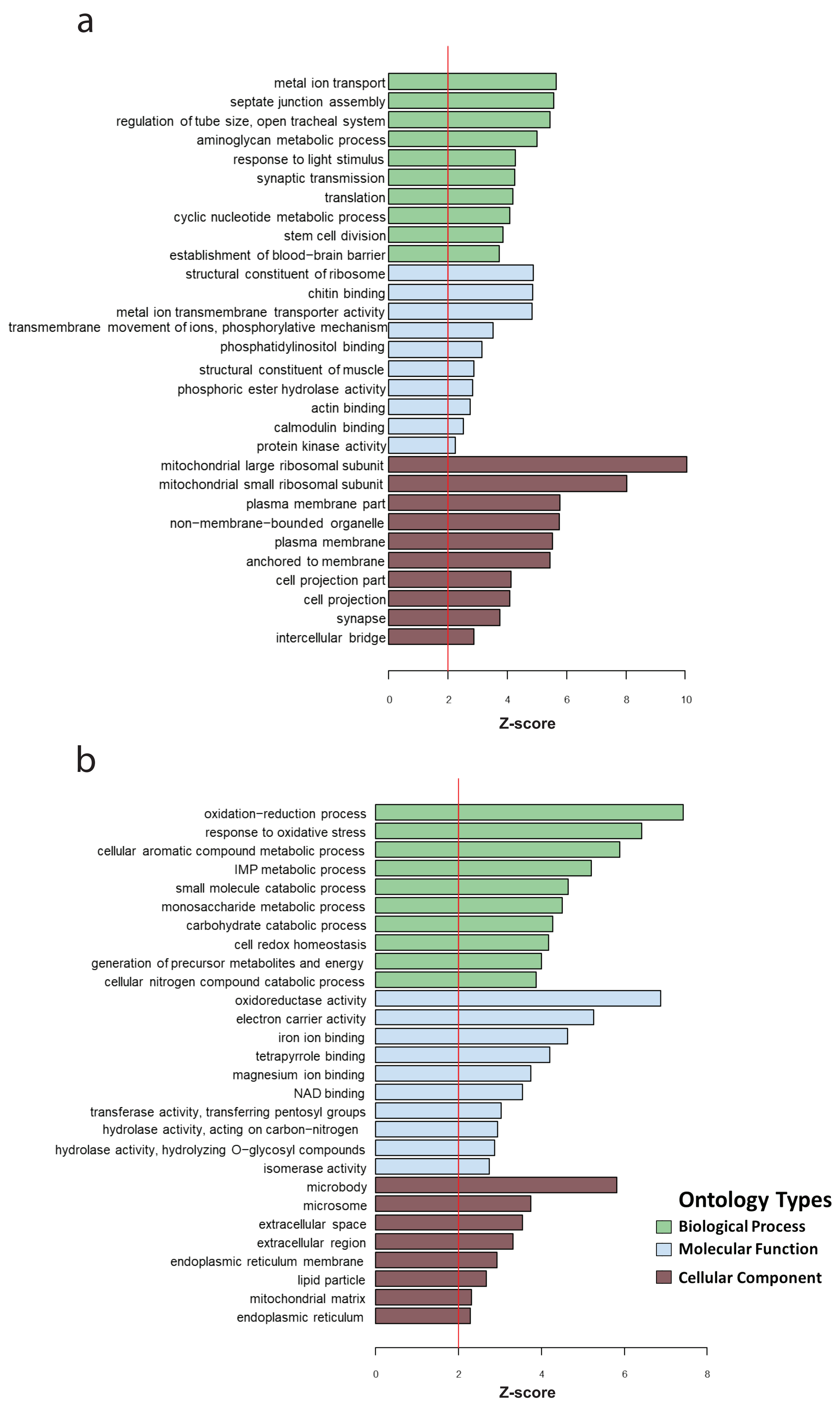

Supplementary Figure 1. Gene ontology analysis of proteins altered by human **α**-synuclein expression**. a, b** GO analysis of proteins (**a**) downregulated or (**b**) upregulated in **α**-synuclein transgenic flies. A Z-score greater than 2 (marked by the red line) is considered as a significantly enriched GO term.

Supplementary Figure 2

a

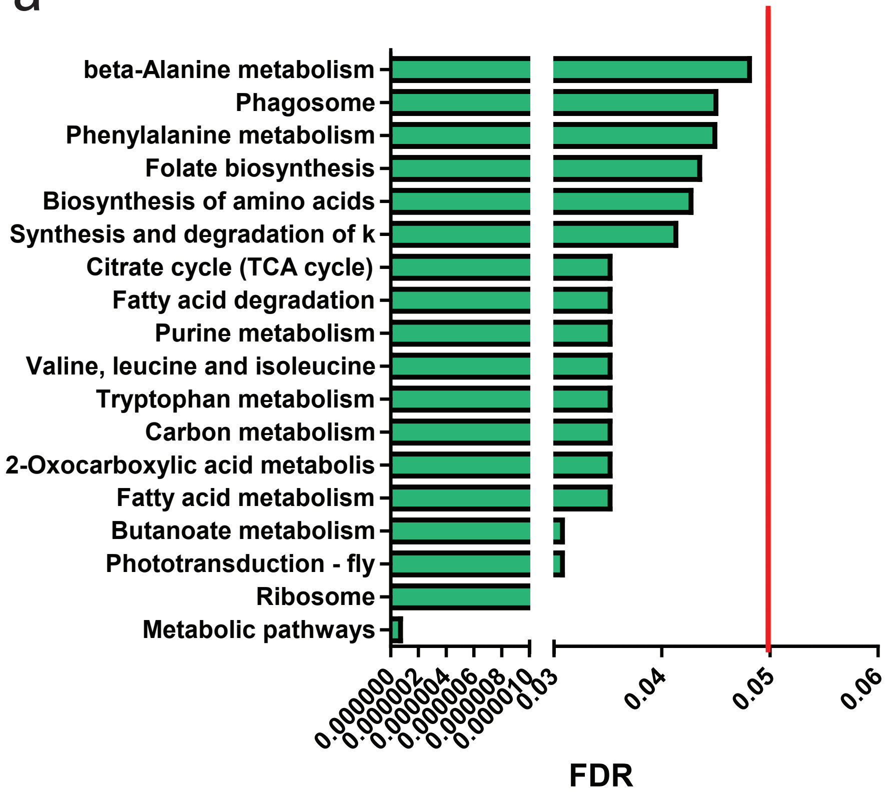

b

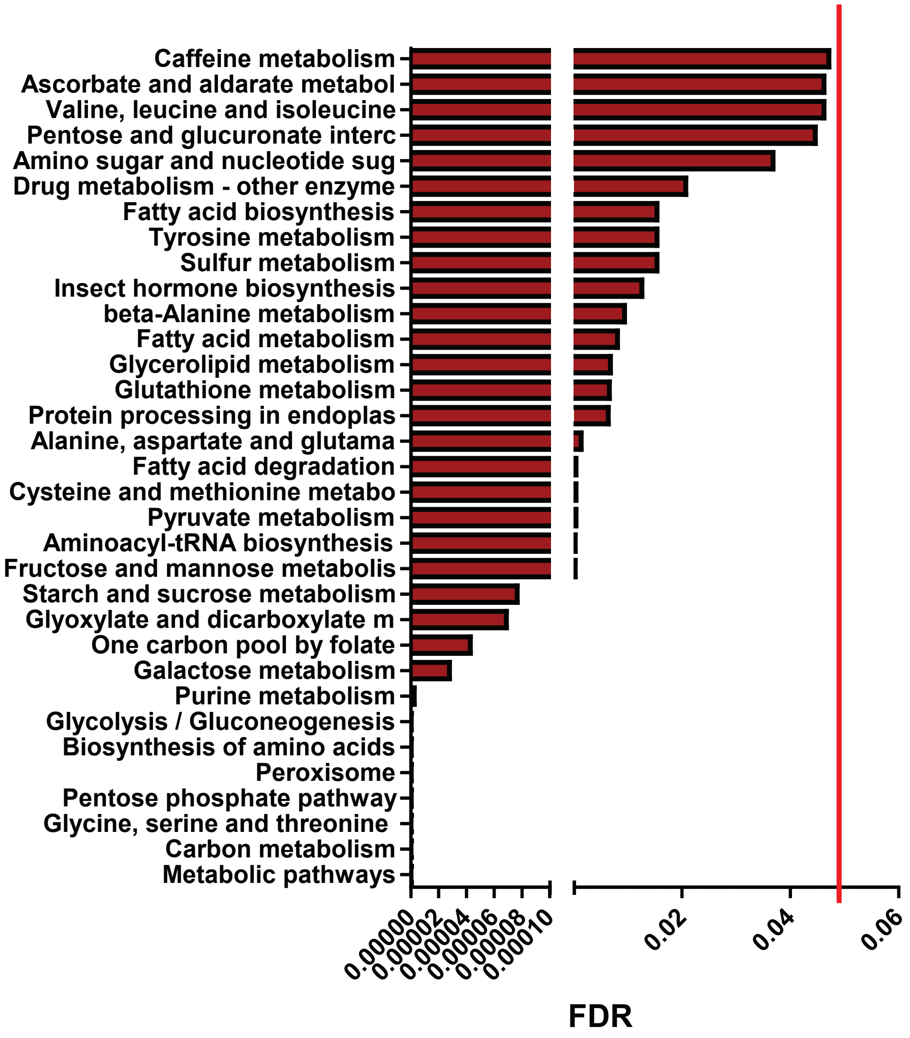

Supplementary Figure 2. KEGG pathways enriched in proteomic analysis of human **α**-synuclein transgenic flies. **a, b** KEGG enriched pathways of **α**-synuclein induced (**a**) downregulated or (**b**) upregulated proteins. FDR: false discovery rate. An FDR < 0.05 (marked by the red line) is considered as a significantly enriched KEGG pathway.

Supplementary Figure 3

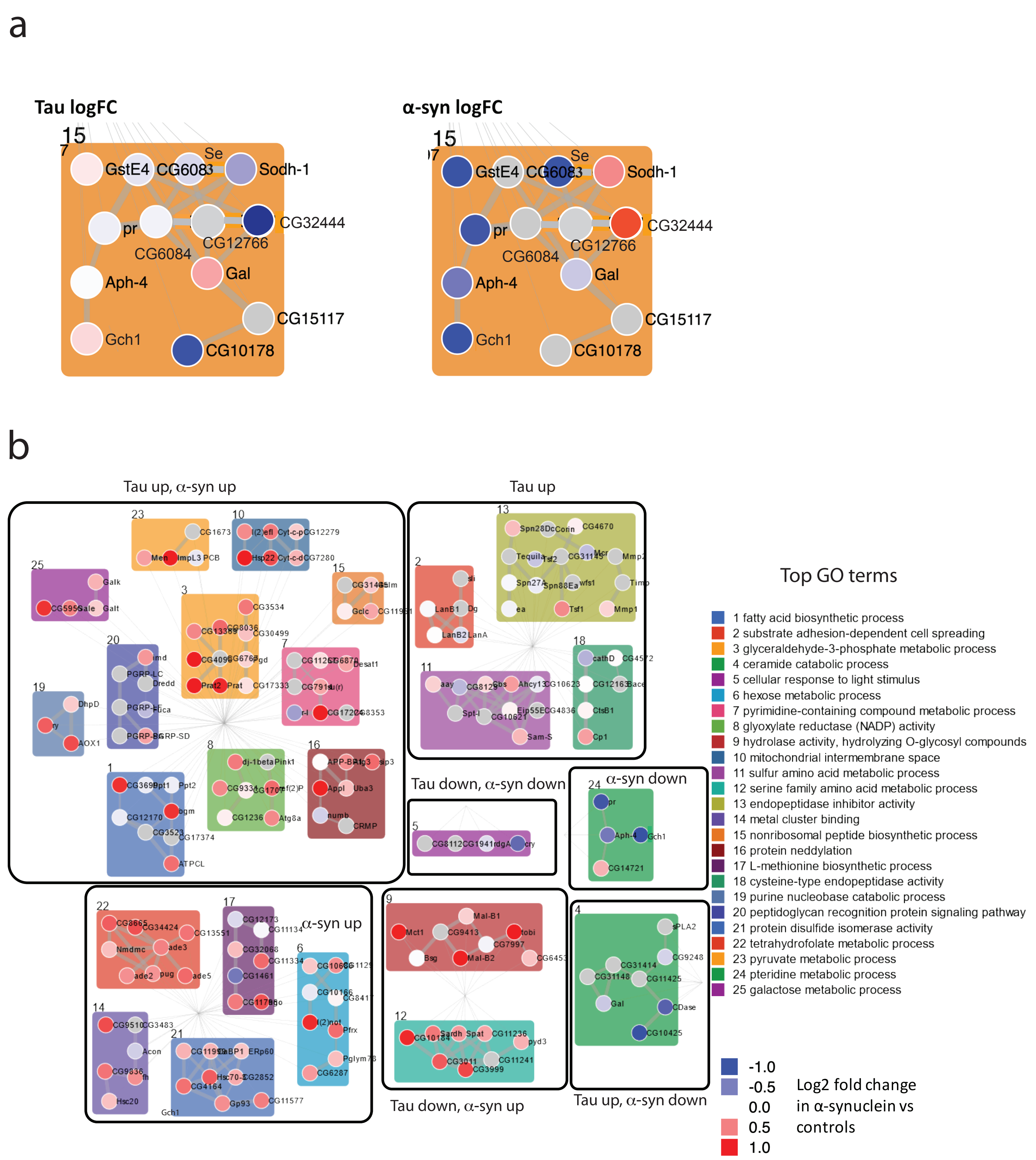

Supplementary Figure 3. Human tau and **α**-synuclein expression induce distinct proteomic signatures. **a** Higher power view of cluster containing Gch1 from Fig. 3c. **b** Network analysis of proteins altered in tau and **α**-synuclein transgenic flies with up and downregulated proteins separately. Clusters are shaded by the color of the most-enriched GO term as defined on the right. Nodes corresponding to the indicated GO term are shaded to indicate degree of upregulation (red) or downregulation (blue). Edges are sized by confidence; edges across clusters are omitted for ease of visualization.

Supplementary Figure 4

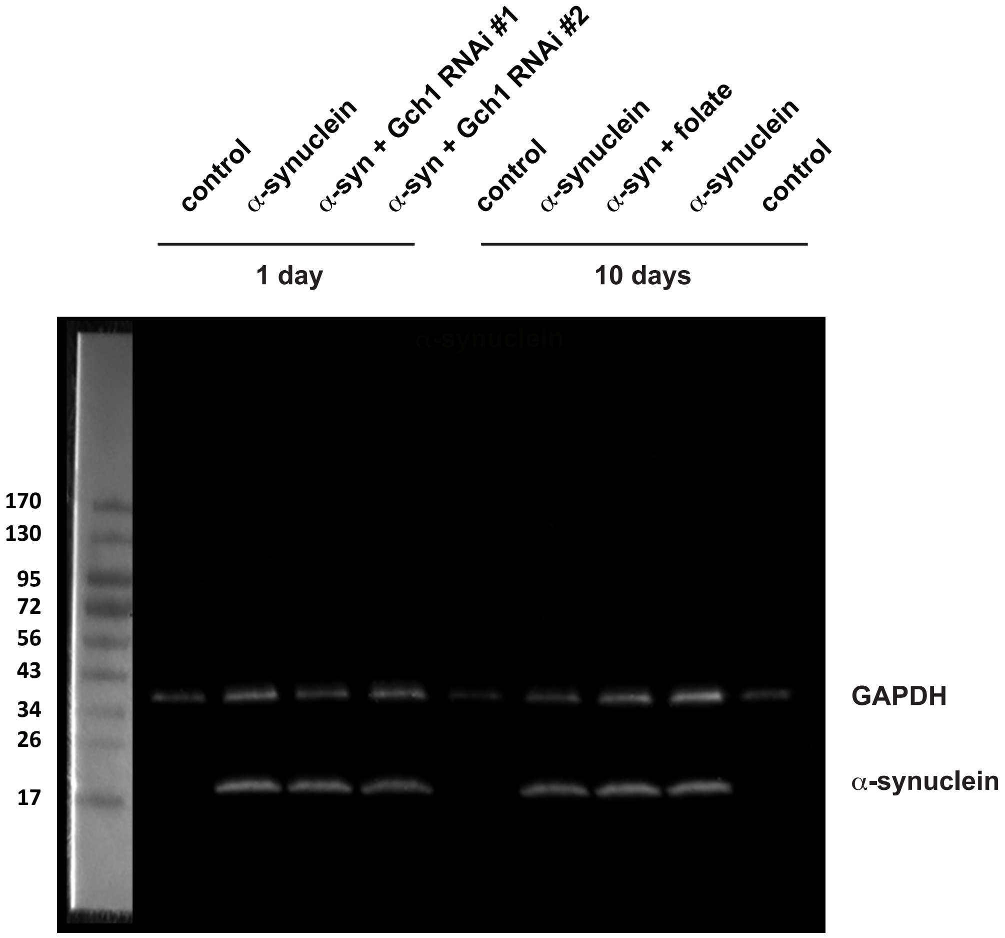

Supplementary Figure 4. Gch1 knockdown or folate supplementation does not alter levels of transgenic human α-synuclein. Western blot analysis demonstrates that neuronal knockdown of Gch1 (right) or folate treatment (left) does not alter α-synuclein protein levels in transgenic flies. Control genotype: *nSyb-QF2, nSyb-GAL4/+*. For Gch1 knockdown experiments flies are one day old. For folate administration flies are assessed after 10 days of treatment. The blot is reprobed for GAPDH to illustrate equivalent protein loading.

Supplementary Figure 5

a

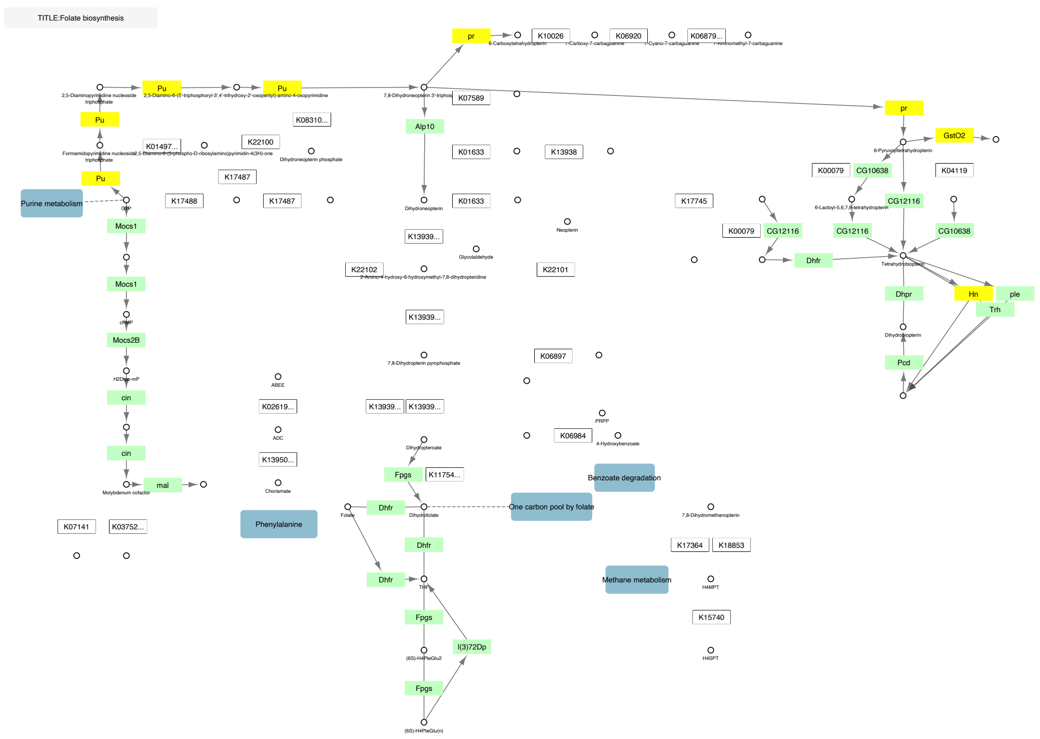

b

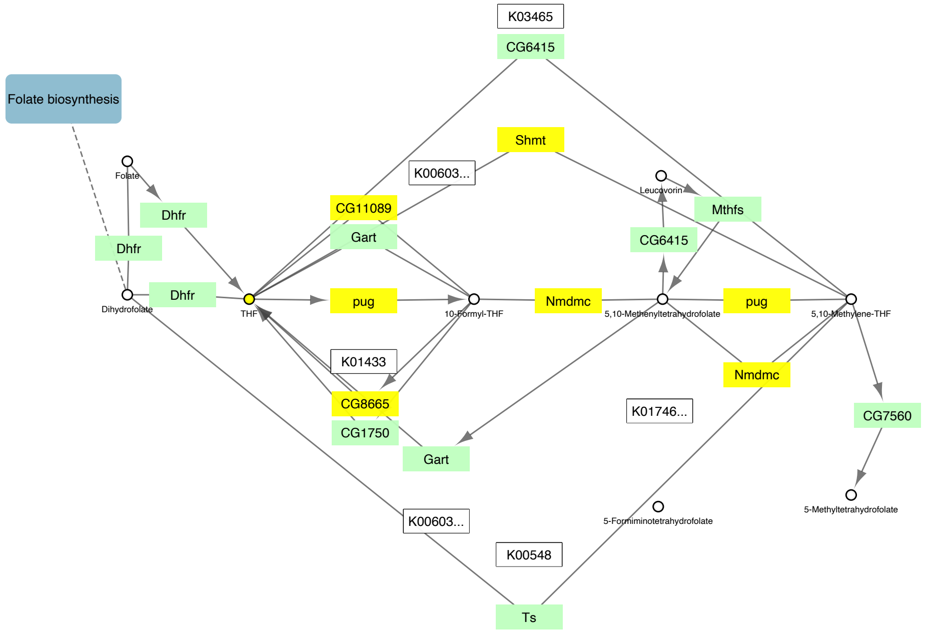

Supplementary Figure 5. Proteins regulated in **α**-synuclein transgenic flies participate in regulation of folate biosynthesis and one-carbon pool metabolism. **a, b** Cytoscape representation of KEGG pathways of (**a**) folate biosynthesis and (**b**) one-carbon pool of folate in *Drosophila*. The yellow highlighted proteins are altered in **α**-synuclein flies. Highlighted proteins are decreased in (**a**) and increased in (**b**). Green is the default highlight color for all proteins in the pathway.

Supplementary Figure 6

a

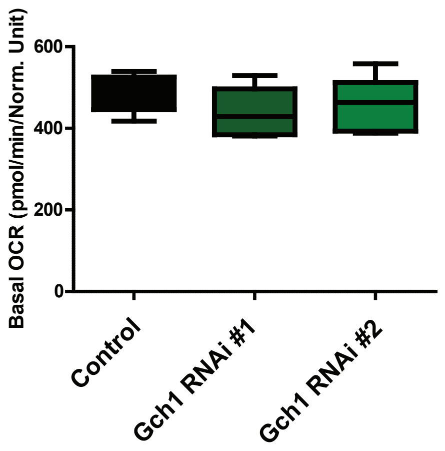

b

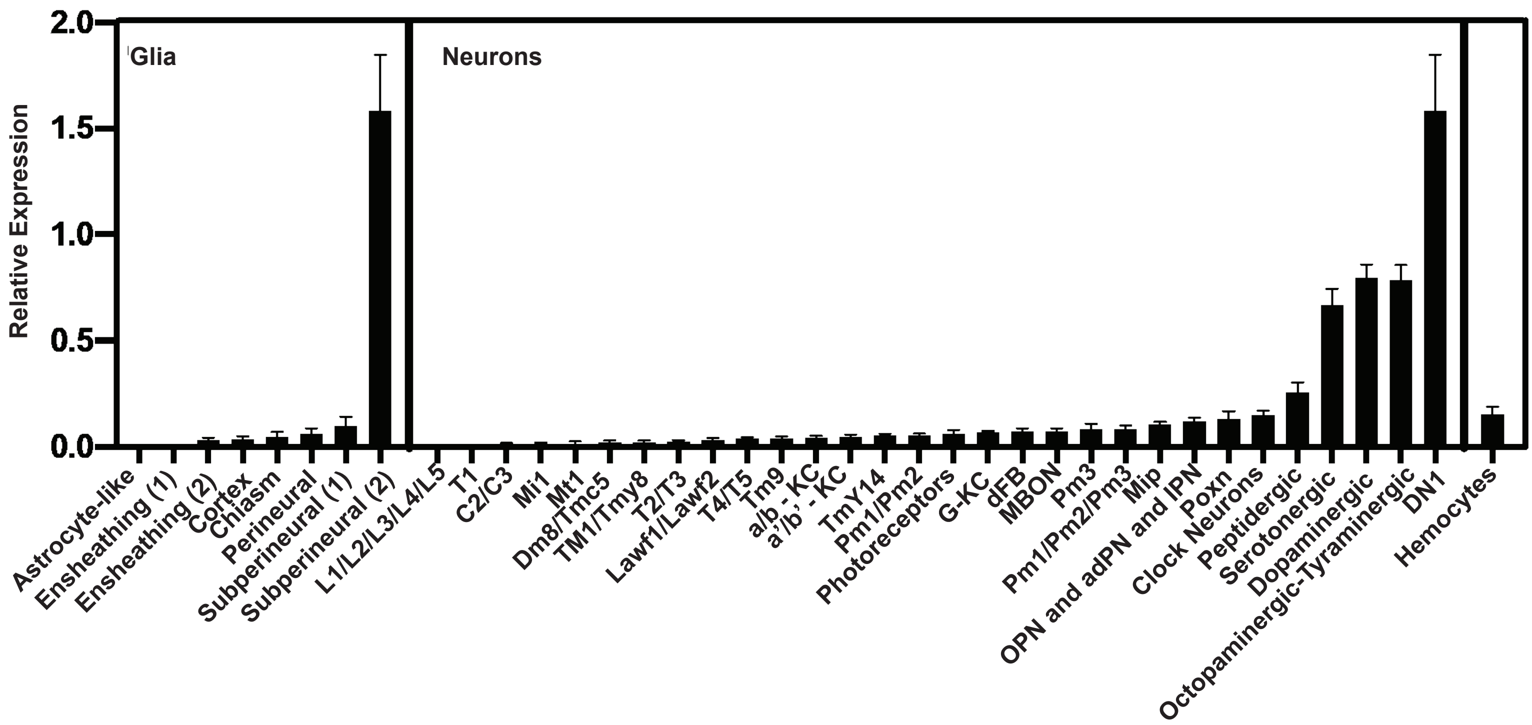

c

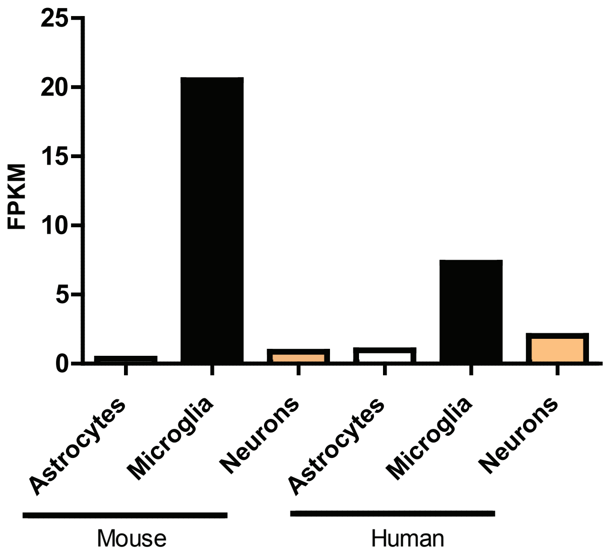

Supplementary Figure 6. Analysis of neuronal and glial Gch1. a Neuronal knockdown of Gch1 using the same RNAi reagents as in Fig. 5, in the absence of expression of transgenic human **α**-synuclein, has no effect on mitochondrial bioenergetics. **b, c** Cell-type specific expression of Gch1 in (**b**) *Drosophila* and in (**c**) mouse and human brain.

Supplementary Data 1. Proteomic changes observed in α-synuclein transgenic flies. Raw and normalized data from control and human α-synuclein transgenic flies are presented (5 control samples and 6 α-synuclein samples).

Supplementary Data 2. List of altered *Drosophila* proteins along with human orthologs of genes associated with Parkinson's disease risk from GWAS studies.
MAGMA identified Parkinson's disease GWAS genes were cross-referenced to the fly proteomic dataset. DIOPT was used to convert the fly proteins to human orthologs. The human symbol, *Drosophila* symbol and DIOPT score are provided.

Supplementary Data 3. Statistics from proteomic changes observed in human α-synuclein transgenic flies. Fold change and statistical analysis from control and human α-synuclein transgenic flies are presented (5 control samples and 6 α-synuclein samples).

Supplementary Data 4. Annotation of altered proteins from single-cell sequencing data to glial, neuronal and unannotated subgroups. Proteins that were differentially expressed (up or down) or proteins that did not change in abundance were mapped to broad cell types (neurons, glia and unannotated) in the fly brain based on a published single-cell RNAseq study.[42] In this study, 87 clusters of cells were identified, and these were collapsed into 3 cell groups listed in the table.
